# Useful lessons for the provision of services in long-term care facilities in South Korea: operators’ experiences illuminate the phenomenon of working with the elderly in the field

**DOI:** 10.1080/17482631.2019.1565238

**Published:** 2019-02-14

**Authors:** Young Ran Tak, Hae Young Woo, Lee Han Yi, Ah Rim Kim

**Affiliations:** School of Nursing, Hanyang University, Seoul, Republic of Korea

**Keywords:** Qualitative research, phenomenological studies, Colaizzi’s seven steps, long-term care, person-centered care

## Abstract

**Purpose**: The aim of this study was to gain a deeper understanding of elderly long-term care facilities by focusing on insights provided by the operators of these facilities.

**Methods**: In this phenomenological study, 10 participants who operated nursing home businesses were interviewed. Of the 10 participants, seven had graduated from a nursing programme and three had studied social welfare.

**Results**: The experiences of facility operators could be organized into four themes: “Starting as a facility operator”, “Dream of an ideal long-term care facility”, “Struggling desperately in practice”, and “Obtaining hope by providing care”. These four themes were divided into 18 subthemes, constituting 96 meaningful statements.

**Conclusion**: Despite the obvious gap between reality and ideals with regard to caring for residents, operators used a person-centred care strategy for the elderly at their facilities. Our findings indicate that, despite diverse barriers and hardships, participants were encouraged when they offered person-centred healthcare services for the elderly under their care, based on a philosophy of a holistic understanding of humans and respect for human life and dignity. Facility operators who are leaders in the practical field should be involved in the decision/policy-making process, to support health and well-being in the elderly in institutionalized settings.

## Introduction

An ageing society is becoming a fact of life in the Republic of Korea (South Korea); South Korean society is fully expected to become an aged society by 2018 and then a super-aged society by 2026, at a globally unprecedented rapid speed (Korean Statistical Information Service [KOSIS], 2011). Such a rapidly ageing population is associated with increasing incidences of geriatric chronic diseases such as dementia and stroke at the social level, and problems such as decreased numbers of females within families, despite the fact that females are traditionally in charge of care. These issues stem from a stronger focus on the nuclear family and the increasing number of working women. Other causes are the increased financial and mental burdens on families due to long periods of caring for elderly family members. For these reasons, elderly care by families is becoming limited. The government enacted long-term care insurance in July 2008 with the objective of establishing the long-term care insurance system at the early stage of ageing, to relieve people’s anxiety about old age, to provide health promotion and stable living for the elderly, who may not be self-sufficient given their age and/or related conditions, to relieve the burden on families, and to improve the overall quality of people’s lives (Jang, 2008).

Many nursing homes established before the law accommodated the elderly and provided them with good physical and mental services, and served to relieve their families of the burden of care, but many also failed to secure specialties as they operated their facilities or failed to realize efficiency in providing services (Sim, 2003). In addition, the criteria for selecting recipients of long-term care services have been determined by the financial circumstances of the elderly, with some depending on government funding for people with geriatric diseases of special classes, such as those in the lower income brackets or elderly residents who live alone (Yun, 2010). In contrast, given that the introduction of the long-term care insurance system for the elderly, which is a public system, is based on social insurance with the principle of a social bond, akin to health insurance, it serves all people who pay into the insurance system and includes a service provision determined by the health status of the elderly person. Therefore, its coverage has expanded from a provider-centred service for those in the lower income bracket to a service for people in general, making it possible to obtain specialized services from trained caregivers. The introduction of the long-term care insurance system for the elderly has positively reduced the financial burden of caring for the elderly, provided beneficiaries’ families with psychological stability, created new jobs (e.g., caregivers), and expanded the welfare instrument business, resulting in a ground-breaking shift towards a system of social insurance.

However, no blueprint has been devised for the elderly health and welfare support system, social discussions and agreements remain insufficient and unfinished, and the reporting system applied in establishing nursing homes, in consideration of limited caregiving personnel and insufficient long-term care facilities for the elderly, has only served to establish the system in its early stages; currently, both individuals and corporations are allowed to establish an elderly healthcare and welfare facility if they meet the criteria set for facilities and personnel assignments based on the Social Welfare Act, consequently lowering the barrier to market entry and drastically increasing the number of institutions providing long-term care services to the elderly (Lee & Yu, 2012). Moreover, as the operation of long-term care facilities for the elderly depends on the market-supply system, care institutions (suppliers) with a profit motive may use several unfair measures to ensure a profit, possibly threatening their financial stability and even their sustainability.

The government initially managed the implementation of the system and later reinforced the criteria for facilities and personnel assignments through a revision of the enforcement regulations of the Elderly Welfare Act, which is considered to be a new law that came into effect in 2008 with the objective of providing improved welfare services upon its initiation. That is, nursing homes established after the enforcement regulations came into effect were required to report their establishment according to the criteria for personnel assignments and facilities based on the new law, whereas those established before that date (i.e., nursing homes established on the basis of the old law) were required to apply stricter criteria for personnel and facilities after a period of grace of 5 years up to 3 April 2013.

Owing to the lack of discussion with directors or managers currently operating these facilities, and in the absence of sufficient explanations during the overall policy implementation process, enforcement of the law applicable after the government’s one-sided pre-announcement of legislation may have confused operators before the long-term care insurance system came into effect and may have made them sceptical of the operation, relying less on the government implementing the policy as their private property became a non-profit entity against their will (Yun, 2010).

In recent years, the number of studies involving care workers and families of institutionalized elderly residents has increased, whereas few research works have investigated the lived experience of operators (facility directors) managing long-term care facilities in the South Korean context. Therefore, this phenomenological study was motivated by the need to provide an in-depth understanding of the meaning that the operators give to their own perception and experience of working with the elderly in South Korea’s healthcare system. As the operators’ practical service adaptability and care management philosophy might be closely related to the quality of care in long-term care facilities, their lived experience or views need to feed into health policy for health and quality of life in the elderly. This study aimed to capture individual viewpoints of this population to deeply understand the phenomena and to explore the meaning of working with the elderly from the perspective of the facility operators. Thereby, relevant solutions which can be useful to those who develop plans or make policy to keep the service public can be suggested.

### Objective

The purpose of this study is to establish a systematic, in-depth exploration of the experiences of operators (directors) of long-term care facilities for the elderly and to assess the characteristics of these organizations according to the aforementioned recent changes following the enforcement of the long-term care insurance system for the elderly in July 2008 and the application of the revised law in April 2013, to provide a clearer understanding of the essence of their experiences.

## Methods

### Design

This study uses a phenomenological method for the in-depth exploration of the diverse experiences of the operators (directors) of long-term care facilities for the elderly and the range of experiences they have undergone after the introduction of the revised law in 2013. Phenomenology emphasizes an attitude of accepting the state of a situation as it is. Nursing phenomena include the relevant individuals’ experiences and their subjective emotions, perceptions, and responses. Therefore, the phenomenological goal is to identify the meanings or essence of these experiences in situations specific to those relevant individuals (Hong, 2011).

### Participants

The participants (*n* = 10), who had been managing long-term care facilities during the period from 2008 to 2013 and were current nursing home operators in both rural and urban areas of South Korea, were purposively selected. The data were collected from the operators through in-depth interviews, which allowed them to verbalize their experiences. The participants’ consent to their participation in the research was also obtained. Snowball sampling (Patton, 1990) was used to select the participants. Ten initial participants were interviewed through snowball sampling, and to secure sufficient data, in-depth interviews were conducted until there were no more new data (saturation).

### Ethical considerations

Before the data collection process, approval for the study was obtained from the Institutional Review Board (HYI-13-104-1) of Hanyang University (Suwon, South Korea). To confirm the spontaneous participation of the participants in the research, they were given a full explanation of its purpose and were assured of confidentiality, anonymity, and their freedom to discontinue the interview if they wished. They were also told that they would not be disadvantaged if they gave no interview, before they submitted written consent to participate. The interviews were recorded with the participants’ consent, and they were informed that the interviews conducted during the course of the research would only be used for the purposes of the research.

The entire volume of the recorded interviews was transcribed into a Hangul word processing file. Personal information related to the participants’ identities was deleted and classified with a separate serial number so that the participants’ identities could not be revealed. Those who wanted a copy of the results were supplied with one.

### Data collection

Data were collected for approximately one month, from 1 to 31 December 2013. The most important instrument in qualitative research is the researcher, who should be trained so as to be qualified to conduct research on a daily basis. The education of the researcher in charge of data collection in this study concentrated on the elderly and dementia, and the researcher participated in several studies at geriatric facilities while undertaking a master’s degree. The author also conducted qualitative research projects at getriatric facilities to explore overall issues in long-term care facilities whilst studying at doctoral level. These activities encompassed phenomenology and grounded theory continuously during the graduate courses, and the researcher has an interest in several issues and phenomena regarding geriatric facilities and specializes in creating valid questions and answers.

A research plan was drawn up to schedule the research before it was initiated. A visit was made to the operator of a long-term care facility for the elderly who was in regular touch with the researcher, to provide a full explanation of the purpose and content of the research and to ask them for their cooperation. The first interviewee was then given an explanation of the criteria for selecting participants and was asked to introduce other participants. Data collection was performed after the researcher personally made a telephone call to each participant to explain the purpose of the research and to receive their consent to participate. The interview time was determined at each participant’s convenience and the interviews were conducted at their preferred site. Because most of the participants preferred their own workplace, the researcher personally visited these facilities and thanked them for participating in the research before the interview. The participants secured access to the quietest and most undisturbed place in the facility. Rapport was established before the interview and the participants were given an explanation of the purpose and methods of the research and all of the answers to any questions they had, creating an atmosphere that could induce full disclosure before the interview started. The interviews started with small talk to make the participants comfortable, and then semi-structured questions were used to allow them to give full descriptions of their experiences, after which supplementary questions were used to allow them to describe their detailed experiences, as needed. The time and place for the interviews were determined according to mutual consent at places other than their workplaces, and these interviews were conducted in a room that only the researcher and the participant were permitted to enter.

Data were collected from each participant through two or three in-depth interviews, and every interview lasted for 1–3 hours. Each in-depth interview was recorded, with the participant’s permission, to prevent data being omitted. While the interview was being recorded, verbal and non-verbal gestures and behaviour of the participants were carefully observed and recorded in a notebook. The interview was transcribed as the participant verbalized on the day, and the analysis was conducted with reference to the verbal and non-verbal expressions recorded in the notebook.

### Instrument (questions)

Interview provide an effective method to use to understand individuals’ lived experiences as they reflect social issues and offer insight into sociological issues. An in-depth, phenomenological interview usually involves open-ended questions; the researcher asks the participants to reconstruct their experiences within the range of the themes, considers their specific experiences and the meanings of the experiences, and conducts an in-depth exploration of multiple issues in the research field. In phenomenological study, the researcher grasps the cues of the participants instead of predetermining the research questions (Ray, 1994). To elicit in-depth experiences for the purpose of this study, we prepared broad, open-ended questions developed around the study aims, such as “Can you describe your experiences in operating a nursing home?”, “What motivated you to operate it?”, and “What have been the challenges and the rewards in your operation of the facility?” This kind of semi-structured interview, generating qualitative data and characterized by open-ended questions, allows the researcher to address the range of issues that need to be covered during the interviews (Chan, Fung, & Chien, 2013). The designed open-ended questions helped us, as researchers, to pursue an area of interest (lived experience of operators of long-term care facilities), while the participants could introduce issues that we had not thought of in advance.

### Data analysis

Colaizzi’s (1978) process was used to collect, analyse, and understand the data, and the collected data were analysed using Colaizzi’s (1978) seven-phase method.

In Phase 1, each participant’s protocols, recorded as raw data, were repetitively read while visualizing the interview situation to gauge the feelings behind their statements, and the meanings were explored using underlined text.

In Phase 2, significant statements were drawn from phases and sentences containing the relevant phenomena among the participants’ statements. Thematic identification was conducted on both within-case and between-case levels to identify the main themes and sub-themes of the facility operators’ experience of managing nursing homes or long-term care facilities. Between-case analyses were used to identify the convergences and divergences among the participants of the core components of the meaning of operating facilities for the elderly as the operators, following within-case analyses, which were performed to investigate the key components of each participant’s experience. During this phase, all authors discussed the themes based on the findings of the initial coding, and thereby the four themes and 18 sub-themes, with 96 significant statements, were formed with the mutual consent of the members in group meetings.

In Phase 3, efforts were made to describe the hidden meanings in the raw data in a more general form to discover what the participants intended to reveal, signified, and wanted to say in the significant statements. During this process, the composition of the meanings was reviewed together with the supervisor experienced in qualitative research, a doctoral degree holder specializing in qualitative research, and a graduate student also studying qualitative research, with the goal of generally restating the 96 significant statements to make the research more reliable.

In Phase 4, sub-themes and themes were devised from the significant statements and restatements. In this process, a comparison was made with the raw data to discover any awkwardness, inconsistencies, or logical jumps. These processes were repeated several times.

In Phase 5, the sub-themes and themes drawn from the statements of the experiences were combined to describe thoroughly the experiences gained in the long-term care facilities for the elderly.

In Phase 6, explanations of each statement, sub-theme, and theme, and of the raw data for the participants, were specified to describe the essential structure of the nursing home operators’ experiences.

In Phase 7, the participants were informed as to whether the results in Phases 4, 5, and 6 were consistent with their experiences as recorded via e-mail. As the data analysis was conducted by the researchers, it was possible that the information had been distorted and filtered. This means that the researchers need to acknowledge that their own interpretations might have an impact on the data analysis. To enhance the trustworthiness of the data analysis, the procedure of validating the results by returning to study participants is secured in the Colaizzi’s data analysis method (Polit & Beck, 2010). This procedure can allow the participants to ascertain whether their answers to any issues had been distorted and can guarantee that the researcher has not misinterpreted the data. In this study, the operators confirmed that “Our experience was correctly interpreted” and that “The results reflected what we had wanted to say.”

### Securing the quality of the research

To secure the quality of the research, Sandelowski’s (1986) four criteria of truth value, applicability, consistency, and neutrality were considered.

The truth value refers to the ability to describe and interpret experiences and to “see if actual phenomena were correctly measured”. Interview records and the analysis results were shown to the participants to determine whether they were consistent with their own experiences.

Applicability refers to the concept of fittingness (transferability), suggesting the possibility of applying the results of this study to other data and the degree that the data can be applied in contexts other than the research situation. Operators other than the actual participants were asked to read the results to note whether the results were significant and applicable through a comparison with their own experiences.

Consistency refers to the ability of any reader or researcher to follow the progress of the research and understand the logic and criteria for determining consistency through the data collection and analysis processes. Thus, the research methods and the process of data collection and analysis were minutely described, recorded, and disclosed.

Neutrality refers to the absence of prejudice in the researcher about the research process and the results. It requires the satisfying of the truth value, applicability, and consistency, and characterizes the objectivity of quantitative research. In this study, the data analysis process was observed and reviewed together with experts and colleagues to secure the quality of this process through feedback from colleagues (Maxwell, 1996).

## Results

The interviewees had the following characteristics. All 10 of the interviewees were female, and their average age (mean ± SD) was 53.88 ± 4.34 years. Seven of them had majored in nursing at college, while three had studied social welfare. They were at the educational level of graduates, of a 3-year college through graduate school or higher, and had operated their long-term care facility for 8.09 ± 2.26 years on average, ranging from 6 to 10 years. They had worked at a geriatric facility for 3.82 ± 4.69 years, ranging from no such experience to 10 years, before starting to operate their own facility. With regard to facility type, seven were long-term care facilities and three were community homes; the former included three urban settings and four suburban settings, and the latter included two urban settings and one suburban setting. The results included four themes, 18 sub-themes, and 96 significant statements, as noted above (Table I).

**Table I.** Themes, sub-themes and meaningful statements.

Theme	Sub-themes	Key meaningful statements
Starting as a facility operator	Gaining an opportunity to operate a facility	It is one of the few entrepreneurial opportunities for nurses.
		I wanted to operate a facility with a nurse’s experience.
		It was a realistic opportunity for occupational independence for a nurse.
	Aptitude as a caregiver for the elderly with dementia	My mother-in-law suffers from dementia.
		I became exhausted while caring for family members suffering from dementia.
		I came to understand family members suffering from dementia.
	Being sensitive to the operation of a facility through practical work	I gained confidence about my line of work while working at a geriatric hospital.
		Finding a solution to caring for the elderly who suffer from dementia leads to a solution to family issues.
		I encountered information concerning long-term care insurance while volunteering.
Dream of ideal long-term care facilities	A home-like, comfortable atmosphere	A nursing home should feel comfortable.
		A nursing home isn’t the resident’s own home, but it could feel like it.
		A nursing home should be a comfortable place of peace and rest.
	A place that is easy to access	A nursing home should be a place where family and/or guardians can easily visit.
		A nursing home should be near a city.
		From one’s home, a nursing home should be easy to travel to.
	A place for person-centred care	A nursing home should be a place of mutual respect between caregivers and residents.
		Care should be tailored to each resident’s needs.
		A resident’s existing abilities should be exercised to the fullest extent.
Struggling desperately in practice	A confused transitional period	It is difficult to earn a reasonable profit due to increased governmental restrictions.
		Directors are changed into salaried employees devoid of passion or interest.
		The government does not recognize private real estate or debt.
	Insufficient information on policy changes	Policymakers have no expertise in elderly care.
		Policies change with each incoming policy administrator.
		I need more open information for long-term care policies.
		There needs to be more information about diagnosis-related groups.
	One-size-fits-all policy without evidence	The implementation of a policy does not take into account the discrepancies in the sizes of facilities and the ramifications of the policy.
		I wish the government would acknowledge the diversity of different care facilities.
		There is no consistency in retroactively applying laws.
	The need for medical fees (benefits) that reflect reality	There is a need for practical fees.
		The government needs to provide funds for the services it mandates are provided for the elderly.
		Due to budget constraints such as diagnosis-related groups, it is impossible to provide a high level of care with specialized services.
	Lack of systematic certification process for facilities	The ultimate goal of inspections should be to increase the level of service.
		Paper-pushing and bureaucracy has taken precedence over actual caregiving.
	Difficulty with personnel management	There are not enough people who want to work as care workers for a variety of reasons.
		Care workers lack professional knowledge.
		Care workers lack a work ethic and professionalism.
	Broken trust relationships with employees	Whistle-blowers make it difficult to operate a facility.
		Caregivers are treated like criminals.
		The policy of “walk on eggshells” is not conducive to a good work environment and is exhausting.
	Loss of self-esteem	I don’t feel empowered to be autonomous.
		Facility operators need to respect the autonomy of employees.
		I feel a loss of self-esteem.
	Negative perceptions of long-term care facilities	It is unfair that other people in the community look upon long-term care facilities and their employees as a group of organized criminals.
		Only poorly run and delinquent facilities are depicted in the press.
		There are no depictions of well-run facilities in the press in any form, whether they are promotions or comparisons with poorly run facilities.
Obtaining hope by providing care	Improvement of residents’ conditions	Residents’ facial expressions have been brighter, and they look more at ease.
		Residents show appreciation and express happiness.
		Residents’ health conditions have improved since their admission.
		Residents’ quality of life has improved.
	Feeling rewarded through work	When familial strife has lessened.
		When burdens on the family have lessened.
		When residents sincerely express their gratitude.
	Having an occupational calling	When I feel that I have found a workplace where I can work without compromising my principles.
		Preventive care leads to diminished costs of elderly care for the government.
		Long-term care in Korea is still developing.
		I gain satisfaction from the idea that I am a job creator.
		Care workers were trained to instil a more professional attitude.

### Theme 1: Starting as a facility operator

The first three sub-themes refer to how the individual started to run a long-term care facility for the elderly. This theme represents a more personal background (endowment) and motivation to work with the elderly with dementia.

#### Sub-theme 1: Gaining an opportunity to operate a facility personally

A long-term care facility for the elderly was one of the facilities that a person, such as a nurse, could personally operate without belonging to any group, before the enforcement of the long-term care insurance system for the elderly. The participants were generally motivated to start operating a facility in consideration of their retirement or old age, and sought to apply their professional nursing expertise gained while they were employed at geriatric hospitals, before the enforcement of the long-term care insurance system. Alternatively, they had begun to take an interest in elderly patients or geriatric facilities while undertaking volunteer work at a religious facility, and started to operate a long-term care facility or community home on the basis of information about the act on long-term care insurance for the elderly.

I was a health centre nurse. Isn’t it hard to work there in old age? Therefore, I thought of my own place where I could continue to work as a nurse without worrying about retirement. Hence, I began this work. (Participant 1)

I was employed as a nurse at a nursing home and was motivated personally to operate it with a nurse’s mind while caring for them as they spent their remaining time in life. I started it out of a suggestion to operate it. (Participant 3)

#### Sub-theme 2: Aptitude as a caregiver for the elderly with dementia

Some participants were motivated to operate a facility owing to their aptitude gained in consideration of their parents’ old age, or they had experienced conflicts with their family while living with an elderly resident with dementia in their own home, and had experienced burnout while caring for this person. Others were motivated to operate a facility because they were fond of elderly patients and took an interest in caregiving.

My mother-in-law has dementia. I quit all of my other jobs to care for her but my family has changed; especially my father-in-law, who got along well with her when she was healthy, and my son also changed gradually. These events were shocking to me. Then, I decided to show my special quality. (Participant 5)

I was searching for a job for realistic independence and aptitude and I found myself fond of the elderly. The elderly with dementia are rather more innocent. (Participant 4)

#### Sub-theme 3: Being sensitive to the operation of a facility through practical work

Some participants began to think of an alternative when they watched families collapse during their personal visits to homes, or made preparations considering the need for long-term care facilities owing to multiple circumstances that presented long-term inpatients with challenges when attempting to return home, when they worked in a geriatric hospital, and some were asked to open a facility personally by the elderly in their lives. Others decided to open a facility as the act affecting long-term care insurance for the elderly was coming into effect, as they became interested in the elderly or geriatric facilities while they were working as volunteers at religious facilities.

I obtained a home-based nurse’s certificate in 2001. I mostly worked at geriatric hospitals and found that long-term inpatients had no suitable place to go after being discharged because their family could hardly care for them at home. Therefore, I keenly felt the need for a nursing home. (Participant 10)

### Theme 2: Dream of an ideal long-term care facility

The second three sub-themes relate to the operators’ views of an ideal elderly long-term care facility. This theme may indicate their ultimate goal of designing and managing friendly, home-like, and accessible facilities which offer person-centred care.

#### Sub-theme 4: A home-like, comfortable atmosphere

As an ideal nursing home, the participants suggested a home-like place where they could maintain their daily life as usual, and not a hospital.

A nursing home is a home, not a hospital for admission and discharge. When a new patient enters it, every other person comes to him or her with care. This is true for employees and for me. We discuss what problems they have and how to solve them. This is similar to a home where the entire family is worried about any member with problems. (Participant 5)

It is not a home they have lived in but it should be a place all members living together can regard as their own home with an alternative family. (Participant 4)

#### Sub-theme 5: A place that is easy to access

The participants suggested a place that is easy for families to visit and caregivers to access as an ideal location for a nursing home. In other words, the elderly felt less lonely in places where their family could drop by on a daily basis and which were adjacent to a school or a market, to feel the presence of other people, as opposed to scenic places requiring scheduled visits.

Scenic places with mountains and a lake are good but difficult to approach and may make the elderly lonely. I think the facility needs to be located so that they can see a school and watch people come and go outside the windows and so that caregivers can drop by anytime when they want to see them. (Participant 2)

Located near home—another home, not real home. A home-like place, though not home they have lived in, which permits an easy visit from home. A place that permits one to drop by like a visit to another home, not necessarily a scheduled visit. (Participant 4)

#### Sub-theme 6: A place for person-centred care

The participants insisted on customized care at the facilities where the elderly were institutionalized. As a desirable facility, they suggested a place where they were given emotional support, listened to carefully, and assisted to make the most use of their remaining ability on the basis of expert observations, not simply to satisfy their basic needs to eat and sleep.

A place where they listen to me in my place, respect me, and meet my needs. (Participant 6)

A place with customized care for each elderly resident. A desirable place where the elderly are allowed to make the most use of their remaining ability, leading a normal daily life while we observe them. (Participant 3)

### Theme 3: Struggling desperately in the practical field

The third theme, comprising nine sub-themes, embrace a wide range of issues regarding limitations of current policies and barriers to running their own nursing homes. This theme indicates the difficult time that the operators went through during and after the transitional period of the adoption of the new policy, when they experienced negative emotions (e.g., loss of self-esteem and dissatisfaction).

#### Sub-theme 7: A confused transitional period

Existing operators have come to have addtional applications which are challenging with what is considered to be the new law since the application of the act on long-term care insurance for the elderly in 2013. As private facilities based on the investment of personal capital were changed into non-profit corporations, private operators felt severely deprived and deflated, and even considered discontinuing their businesses.

The business was coded as a non-profit. This system is actually operated with social insurance, isn’t it? I think it is right in principle, but private ones were induced to participate due to the insufficient national budget at its initial stage; the coding as a non-profit was not our choice but was directed suddenly, and I’m somewhat confused. (Participant 9)

At the initial stage of the policy, they let us establish a facility with personal finances according to a free economy and now they changed it into a corporation and made directors wage-earners. They need to make some criteria and prevent one from following them, but even good facilities …. (Participant 5, sharing the opinions of Participants 1, 4, 6, 7, and 8)

#### Sub-theme 8: Insufficient information on policy changes

Participants who failed to obtain accurate information when the government implemented the policy demanded the disclosure of information and complained of the one-sided direction in the implementation of the service.

I hope to be given basic directions for the policy. Then, we can gain the big picture when operating a facility, but we have no idea about anything. Nobody knows. (Participant 5)

We need information disclosure. We have no idea about any detail each year. It is unilateral. Nobody has asked for our opinions. (Participant 8)

#### Sub-theme 9: One-size-fits-all policy without evidence

The participants indicated the need for a uniform application of policies, despite the fact that there are diverse facilities in South Korea. Even policies on community homes or long-term care facilities were differentially applied without any definite basis.

In the policies, the same manual is applied to both small and large facilities. They are made to operate in the same system. (Participant 5)

The parking lot contained in the total floor area for downtown buildings is now included in the total floor area. However, suburban cases have no such advantage according to the new law. (Participant 10)

#### Sub-theme 10: The need for medical fees (benefits) that reflect reality

The operators experienced limitations of service bound by the diagnosis-related group (DRG) payment system. However, the government required the provision of practical benefits or services for beneficiaries through the approval of various non-benefit items, as this system could hardly provide a service any longer.

What is most necessary is a practical benefit. It is not appropriate when used to meet all of the service-related requirements of a corporation. However, a corporation demands more ideal services. We can’t provide all of the services without supplementation to the current benefit. (Participant 9)

#### Sub-theme 11: Lack of systematic certification process for facilities

The participants admitted the need for certification assessments of facilities, but felt dissatisfied with the assessments based on documents and suggested several alternatives.

The ultimate goal of the current assessment is to give good care and improve the quality of service, isn’t it? However, I think the consultant inspected other facilities. He asked me how such a gap could be found among those facilities at the same A grade. This occurs because assessments are made on the basis of documents …. (Participant 7)

We hope the assessment process will be changed so that we can see the current situation—not the way of notifying of the day of the assessment in advance but allowing us to be prepared all the time. It is desirable to visit a facility one or two hours in advance. I think this will motivate the facility to be prepared all the time in practice. (Participant 9)

#### Sub-theme 12: Difficulty with personnel management

The participants experienced difficulty with the supply and demand of care workers, and their poor levels of expertise. Moreover, they experienced difficulty with personnel management owing to the high turnover rate and the poor professionalism among employees.

Care workers in charge of the elderly may have no expertise because there is no course for professionals who will care for the elderly at nursing homes. (Participant 3)

They don’t take their job seriously. They need to know that they don’t just wash, feed, or put old women to bed …. (Participant 2)

#### Sub-theme 13: Broken trust relationships with employees

When the government monitors the operation of the facility, a whistle-blowing system for employees at long-term care facilities for the elderly operating with social insurance can cause the operators to withdraw further, as higher walls of distrust are built among employees.

The issue of whistle-blowing. This is so … This work is not to make shoes or other things but to serve people and the tasks are based on trust. The facility cannot engage in irresponsible operation, but is there any other way of screening? Is this inevitable? (Participant 10)

The issue of whistle-blowing. You can think we may not commit a fault, that’s all, but you know: it can be misused by employees. How can there be a perfect person or facility? We need to work together in mutual trust but we read everyone’s face because of this system. (Participant 5)

#### Sub-theme 14: Loss of self-esteem

The participants had no autonomy, had lower self-esteem, and became more withdrawn because they needed to provide fixed services to the patients.

We have no autonomy and find our self-esteem lowered. (Participant 7)

I think it is necessary to respect the operators’ autonomy. Then, they would develop more positively. (Participant 9)

#### Sub-theme 15: Negative perceptions of long-term care facilities

The participants experienced that there was a negative view of all facilities through the reporting of negative facilities rather than positive ones, and indicated social prejudices concerning the facilities and instances of local selfishness.

They see us as if we are a criminal group. Media exaggerate the issue. I doubt they believe that they can change the policy as they want. I feel sorry that they make all the facilities appear in this light. (Participant 5)

We have many civil grievances. For example, we had a puppy as part of cognitive therapy for the elderly with dementia and got a complaint about the barking. They never complain of any dog barking in other places. (Participant 1)

### Theme 4: Obtaining hope by providing care

The last three sub-themes relate to the positive aspects of caring for the elderly in institutional settings. This theme may indicate a philosophy of care that the operators hope to provide in nursing homes, regardless of internal or external threats.

#### Sub-theme 16: Improvement of residents’ conditions

The participants felt joy and that the elderly people were thankful for their care, as they saw improvements in their facial expressions, health, and quality of life.

“I have no worries”, they say with a peaceful expression. “I have no worries here, but they have problems”. Then, I find it worthwhile because they think of this place as their home and feel comfortable. (Participant 8)

On their last holidays, they took lots of medicine for three or more days but none of them spent two nights there. Everyone returned the next day because they felt uncomfortable. They wanted to come right back after breakfast. (Participant 5)

#### Sub-theme 17: Feeling rewarded through work

The participants could see the faces of the elderly brighten and saw that they felt more comfortable, contrary to concerns, as they stayed at the institution, and found that their family felt relieved by having a lighter burden.

“Now it’s a family”. We know from their eyes when they thank us sincerely. (Participant 2)

There are numerous family crises, aren’t there? Elderly are institutionalized here and we see their problems solved. (Participant 8)

#### Sub-theme 18: Having an occupational calling

They experienced thankfulness and joy for being able to provide care to the best of their abilities, believing that this was a good place for the elderly to spend the last days of their life. Regarding the issue of personnel management, the operators made efforts to improve professionalism and expertise through their own training programmes. During the transition period, when the old law was replaced with the new law, they undertook positive operations while they searched for a steady method to change and adapt, cooperating with one another to communicate with policymakers.

I find the greatest worth and feel good when I wash the face and body of the diseased with my hands and dress them with new clothes. (Participant 3)

The employees have been allowed to work flexibly. Fixed work hours and optional shifts which they can select as they want permit housewives not to be bound strongly by the tasks, and some think of it as an advantage because they can finish their personal chores before work. (Participant 10)

## Experience and growth process of nursing home operators

**Figure 1.**
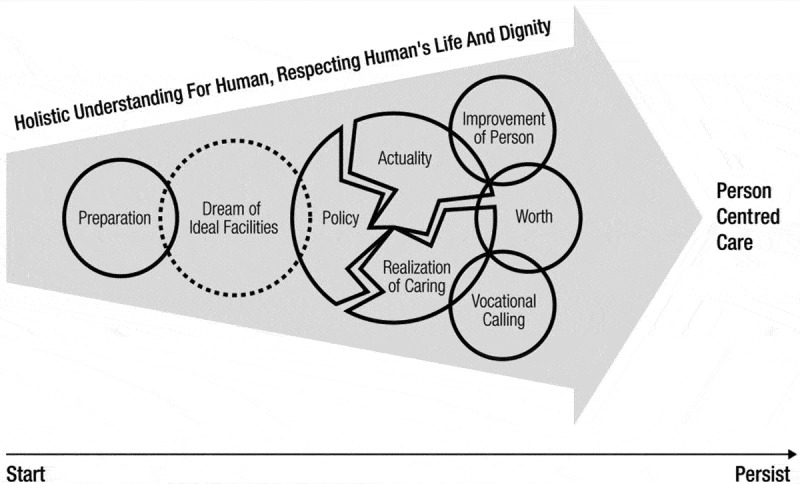
Experience and growth process in operators of long-term care facilities for the elderly.

This study provides an understanding of the entire process experienced by the operators of long-term care facilities for the elderly, from their initiation to their current operation, on the basis of a temporal stream. Having made decisions and gained direct and indirect experience working at other facilities, they experienced changes when they personally operated a facility. Figure 1 presents a possible model showing how the operators of long-term care facilities for the elderly may change and grow over time. Specifically, regarding the opportunity to operate a facility considering the enforcement of the act on long-term care for the elderly, participants with direct or indirect experience of working with the elderly achieved a new start as operators. They made a blueprint of the most ideal facility based on their ideas, research, and experiences, and personally operate their facilities with the final goal of realizing person-centred care. However, they became confused by the application of the revised act on long-term care for the elderly over time. They experienced fundamental regrets concerning the operation as a result of problems such as the difficulty in reflecting the policy due to the gap between the policy and the reality of actually operating the facility, issues of personnel supply and demand and qualitative security, and the reduced trust due to socially negative perceptions. Such struggles and difficulties served as practical limitations to realizing person-centred care. However, the participants in this study continued to operate their facilities despite such practical difficulties because they gained new hope through their efforts to realize their own person-centred care. In other words, they continued their operations not only because they saw inpatients improve but also because they saw family crises relieved, ultimately obtaining a vocational calling.

## Discussion

This phenomenological study was conducted to determine the experiences of facility operators and to find out what the experiences meant according to institutional changes during the transitional period (after the enforcement of the long-term care insurance system for the elderly in July 2008 and the application of the revised act in April 2013). This study is significant in that it directly describes the gap between reality and the system that operators experience when actually operating the facility, and the realistic problems that arise when attempting to attain the ultimate goal of person-centred care, from the operators’ perspectives. Whereas previous studies in nursing homes based on the enforcement of the act on long-term care for the elderly determined the quality and quantity of care services for the elderly with the implementation of a simple policy (Choi et al., 2010; Park, Cho, Nj, & Seo, 2010) or directly indicated inpatients’ experiences of medical demands from nursing employees’ perspectives (Park, Suh, & Lee, 2013), the results of this study not only indicate problems with the current system, before the enforcement of the act on long-term care for the elderly up to the present with the revised law, from the perspective of nursing home operators, but also provide in-depth descriptions of a wide range of experiences related to problems encountered by the institutionalized elderly and their families.

For theme 1, starting as a facility operator, nurses already felt the need and sensitivity given their work at a facility, and had operated their welfare facility or made numerous preparations to operate the facility before the introduction of long-term care insurance for the elderly. Others began to operate their nursing homes given that social welfare majors were allowed to operate such facilities as an institution under certain conditions (Article 22.1 of the Elderly Welfare Act) upon the introduction of the policy. This result means that the motivation of nurses or social workers to start to run long-term care facilities was seen as natural, with good intentions (such as helping older people and their families based on their clinical knowledge and skills), regardless of any profit or loss.

With regard to theme 2, an ideal nursing home as perceived by the participants is not a type of hospital but a home-like facility where the elderly can be given customized care at a location near their homes so that their families can visit with ease. This is consistent with Simmons (2011), who found that long-term care facilities for the elderly with dementia, who cannot complain about the quality of the care, should not only have staff who watch out for physical or mental abnormalities but also have skilled employees to provide person-centred care. This is in contrast to Verbeek, van Rossum, Zwakhalen, Gertrudis, and Jan (2009), who published inconclusive results about small, home-like facilities and suggested the need for further research on appropriate facilities, giving consideration to all users, their families, employees, and expenditures. Most facility operators in this study pursue an ideal institution of high-quality care for the elderly providing an easily accessible and familiar environment while working with elderly inpatients.

For theme 3, the participants who had had great expectations and ideals encountered a barrier to reality when operating their facilities, and thereby faced a struggle of limitations and problems with current policies. These operators ran their facilities before the introduction of the policy and either failed to adapt the facility to the revision of the law in April 2013 or felt confused about the retroactive application. As Kim (2006) emphasized, the duality of allowing those hoping to make a profit through the sale of facility services to develop the service and operate the facility through the market, and those targeting social welfare to realize public welfare, South Korean policy as it pertains to long-term care facilities fails to recognize the diversity of facilities and differentiate them accordingly, but is uniformly applied, which leads to difficulties with communication between the operators and the government or policymakers.

One of the difficulties encountered by operators was the management of care workers, who form the majority of nursing home staff, probably because the introduction of the policy led to a large number of care workers in its initial stages, causing lower quality and poor supply-and-demand levels in some regions owing to the production of a number of care workers in a short time with lack of completion of continuing education system and verification of their levels of expertise. While the government-rewarded system of whistle-blowing, with the purpose of realizing qualitative improvements in the facilities and reinforcing regulations, has some positive effects in cleaning up this sector, it should be applied carefully because it can lead to unfair reports and present excessively negative perceptions of such facilities. Nursing facilities with responsibility for people’s lives must improve the quality of their services and maintain a management team with the objective of improving the quality of life for the elderly. However, excessive limitations on benefits or non-benefit items due to the DRG payment system may lead to minimal levels of service as set by policy and a failure to provide even necessary services. In addition, many families have a poor understanding of nursing homes and demand diverse services, including excessive medical treatments, from the facilities. However, the care workers are reluctant to provide that level of care to institutionalized people, given physical and financial considerations. For this reason, it is difficult to provide a diversity of individualized or customized long-term care services under the current system (Park et al., 2013). Therefore, the government needs to consider a reorganization of policy (e.g., the rewards system of whistle-blowing, one-size-fits-all policy, and DRG payment system) to ease the labour shortage and to reflect difficulties on a practical level, while providing sustainable education and training to those who are charge of caregiving in long-term care facilities.

In regard to theme 4, despite the diverse difficulties in the practical field, the operators could continue to operate their facilities by improving the general quality of life of the elderly, by observing them in the last days of life, and by feeling value in resolving the issues of the burden on and collapse of some families through the wholly personal care they provided. The long-term care insurance system for the elderly has caused a paradigm shift in elderly welfare in South Korea and has caused existing and emerging facility operators to adapt to the continuously changing system, presenting challenges through these changes and adaptations owing to the differences from the existing type of operation. If governmental policy is intended to improve the quality of life of the elderly and relieve the families’ physical and financial burdens through long-term care insurance for the elderly, the operators’ holistic views should be reflected in the policy, in that their roles may be significant with regard to the selection and constant use of long-term care facilities by caregivers (Hong & Son, 2007). Considering that customized institutionalized care services are provided and recognized as goals of nursing homes in advanced countries faced with the problems of the elderly due to ageing (Lim, Choi, & Lee, 2012), it is also necessary for South Korea to develop methods to enhance the operational efficiency and improve welfare performance on the basis of operators’ experiences in running nursing homes (Hong, 2008).

This study is significant in that it explored the phenomena of the facility operators’ experiences. The results showed a specific shift in the welfare paradigm for care facilities in South Korea through these experiences. While operation was based on autonomous service provision according to the characteristics of each facility before the introduction of the system, operation based on a conscious shift in the mindset of the facility management team is necessary, as the introduction of the system has led to a need for unique service provisions to meet the policy requirements.

## Conclusion

This study undertook phenomenological research to explore the essence of the experiences of operators of long-term care facilities for the elderly in South Korea. The participants started up as operators when they found an opportunity to operate a facility based on their numerous instances of direct and indirect experiences with the elderly. Although they applied several plans and methods to operate the most ideal facility they had envisioned when operating the facility, they experienced difficulties in operating the facility owing to policies that were isolated from reality, as well as difficulties in the procurement and management of personnel, lower self-esteem due to socially negative perceptions of their facilities, and even fundamental regret over having opened the facility. Despite such difficulties, however, they operated their facilities on the basis of their understanding of people, especially the elderly, and, ultimately, continued to operate their facilities while providing person-centred care.

In the established current health system, which allows the administration to determine routine care or services, the facility operators struggle to offer good quality of care, reflecting the needs of the different patients and families in different contexts who have responsibility for and authority over admission to a long-term care facility. While institutionalization of the elderly in such a facility is gradually becoming regarded as a natural right, given that the introduction of the system requires the public to pay insurance fees as well as fees for using the service, efforts should also be made to establish good relationships with the communities in which they exist. Finally, this research on facility operators’ understanding of holistic health and human life, and knowledge of sociopolitical issues in older people, may assist health providers, educators, and policymakers in preparing them to take part in decisions about the long-term care insurance system. It is necessary for the operators to enter in-depth discussions on how they will improve the quality of services in these facilities, along with minimizing the financial burden on the institutionalized elderly people and their families, in search of an agreement with the government in meetings intended to promote their health and well-being.
